# Educational interventions to improve women’s preventive behavior of sexually transmitted infections (STIs): study protocol for a randomized controlled trial

**DOI:** 10.1186/s13063-022-06663-5

**Published:** 2022-09-02

**Authors:** Afsaneh Karami Juyani, Fatemeh Zarei, Shamsodin Niknami, Alireza Haydarni, Raziyeh Maasoumi

**Affiliations:** 1 Department of Health Education and Health Promotion, Tarbiat Modares University, P.O. Box: 14115-331, Tehran, Iran; 2 Department of Reproductive Health, School of Nursing and Midwifery, Tehran University of Medical Sciences, Tehran, Iran

**Keywords:** Educational: https://www.ncbi.nlm.nih.gov/mesh/?term=Educational+intervention, Primary prevention: https://www.ncbi.nlm.nih.gov/mesh/68011322, Sexually transmitted diseases: https://www.ncbi.nlm.nih.gov/mesh/?term=Sexually+Transmitted+Infections, Women: https://www.ncbi.nlm.nih.gov/mesh/68014930, Instructional System Design (ISD) model

## Abstract

**Background:**

Sexually active women aged 18 to 48 are within the population at risk for acquiring sexually transmitted infections. Some STIs can cause serious complications in women’s reproductive health. Accordingly, this practical randomized trial aims to evaluate the effect of an interventional education based on the ISD model on improving preventive behaviors for Iranian women.

**Methods:**

Women aged 18–48 years that refer to Tehran Municipality Health Houses will be invited to join the study. Recruitment will continue until a sample of 150 women participants. The study will be conducted using a mixed-methods protocol in three phases. In the first phase, women’s educational and learning needs about STIs will be identified using a qualitative approach. In the second phase, the results from the qualitative approach will be used to design a training program based on an ISD model. The educational intervention will be performed in the third phase. Participants will be randomly allocated into two groups: (1) the intervention group and (2) the control group. Data will be collected using STI Four-Scale of Preventive Behaviors at baseline, immediately, 1-month, and 3-month follow-up assessments. The impact of the intervention on the promotion of preventive behaviors from STIs will then be evaluated.

**Discussion:**

This study provides an educational program for empowering and promoting behaviors that prevent STIs. If the designed interventions in the present study are effective, it has practical potential to be generalized for Iranian women at risk of STIs.

**Trial registration:**

ClinicalTrials.gov IRCT20200602047638N1. Registered on 22 May 2021 with the IRCTID, V1.0.

## Background

In both developed and developing countries, sexually transmitted infections (STIs) are regarded as one of the most serious public health issues [1] More than a million people are infected with STIs every day around the world, and 50 million are infected with one of four curable sexually transmitted bacterial infections, namely chlamydia, gonorrhea, syphilis, and trichomoniasis [2]. The World Health Organization (WHO) estimated in 2008 that the total incidence of four preventable STIs in the Eastern Mediterranean region was 26.4 million [3]. Although the risk of HIV infection and death has decreased in most areas of the Middle East and North Africa, the virus’s prevalence is increasing. The actual prevalence of STIs in Iran is much higher than official data and records indicate [1, 4].

The Ministry of Health and Medical Education in Iran presented alarming statistics on the rate of STIs (such as AIDS) and their rate of transmission through sexual contact. According to reports, sexual transmission has increased from 10 to 21%, and 38.9% of the 28,000 cases registered in 2013 acquired it through unsafe sex [5]. Furthermore, 1700 cases of gonorrhea and 5500 cases of chlamydia have been reported in Iranian men and women. These infections were found to be slightly more common in women than in men [6].

Some STIs can cause serious complications, including pelvic inflammatory disease, infertility, ectopic pregnancy, cervical cancer, neonatal death, or congenital anomalies. Meanwhile, these infections can facilitate the spread of bloodborne diseases such as the human immunodeficiency virus (HIV) and hepatitis B virus via sexual contact [7]. In addition to the sexual route, blood product transfusion, mother-to-child breastfeeding, intrauterine, and delivery are all known ways for some STIs to be acquired [8]. STI complications have a disproportionate impact on people of all ages, with significant consequences for women of reproductive age. Women are biologically more vulnerable to such STIs than men, and they are more likely to experience problems as a result [9]. Women who engage in sexual activity are at risk for STIs [10, 11].

Current efforts to prevent the spread of STIs are insufficient and despite significant efforts to identify simple interventions for reducing high-risk behaviors, changing behavior remains a complex challenge [3]. Several reasons highlight challenges in sexual health education in particular STIs. The results of a review study show that, in general, Muslim women have poor knowledge about the signs and symptoms of STIs, how to prevent, diagnose, and treat them, in addition to many concerns. wrong concept. Negative attitudes towards people living with HIV/AIDS are widespread, and this attitude is strongly influenced by misconceptions and inadequate knowledge. Infected women tend to face more blame and judgment than men [12]. The fact that the mere existence of sexually transmitted infections (STIs) in Iran and other Muslim countries is considered taboo by the government and the public has created a state of denial, which is what some call “a health crisis behind the veil.” [13–15]. Furthermore, individuals’ access to effective resources has been hampered in many developing countries due to negative attitudes toward sexual health education, ineffective communication skills, insufficient educational materials, and a lack of knowledge [16].

Despite being prohibited in the religion of Islam, premarital and extramarital sexual relationships are by no means non-existent, and the scarce literature dedicated to sexual practice in Iran confirms this [16]. Moreover, the incidence of premarital sex has increased over the years [17, 18]. Iranians’ sexual health may be jeopardized as a result of receiving incorrect sexual information from the internet and other sources [19, 20]. Furthermore, discussing STIs is a cultural constraint and taboo [21].

From 2010 to 2013, the third national program on AIDS and STIs in Iran included four STI strategies such as education, sexual transmission prevention, treatment, and strengthening of the epidemiological care system with data management [22]. Educational intervention is one of the most effective strategies for behavior change [23]. The educational approach’s goal is to provide people with the knowledge, information, and skills they need to adopt healthy behaviors. Also, behavioral approaches use preventive strategies to encourage individuals to adhere healthy behaviors [24]. Globally, the prevention of high-risk behavior and unprotected sex, as well as the promotion of healthy behavior, has been identified as the most effective solutions for STI prevention [25]. The timely and rapid diagnosis of disease, complete and effective treatment, education on prevention and risk reduction, and encouraging the use of condoms are some of the principles that can control and cure STIs [26].

In general, health-related behavior and its determinants are defined as “personal attributes such as beliefs, expectations, motivations, values, perceptions, and other cognitive factors.”; personality traits, including affective and emotional states and characteristics; and manifest behaviors, actions and habits related to the maintenance of health, the restoration of health and the promotion of health” [23]. A growing body of literature specifies that behavioral interventions with clear conceptual frameworks or theories are more effective than those without [27]. Measuring the use of behavioral theories/models is a key step in supporting theory/model-based behavioral interventions [28, 29]. In line with this, several studies have shown that theory-based training programs are effective in improving sexually transmitted disease prevention behaviors in Iranian women [30–32]. Given the above, it seems that educational interventions based on theories that consider the learning needs of the target group will be effective in promoting preventive behaviors of sexually transmitted infections in at-risk women. Therefore, this study mixes methods will be used to design and evaluate an educational program to promote preventive behaviors of STIs among at-risk Iranian women.

### Hypothesis

The main aim is to assess the impact of an educational program based on the ISD model on improving preventive behaviors for STIs Iranian women. Intervention effects will be examined at 4 months. The research hypotheses are as follows:

1. The intervention group will show higher preventive behaviors for STIs than the control group measured by mean scores.
2. The intervention group will show STI knowledge more than the control group measured by mean scores.
3. The intervention group will show STI vulnerability than the control group measured by mean scores.
4. The intervention group will show STI prevention self-efficacy more than the control group measured by mean scores.
5. The intervention group will show STI prevention intentions more than the control group measured by mean scores.

### Trial design

The evaluation design is a parallel, randomized controlled trial, with two arms and with a 1:1 allocation ratio. The intervention arm will receive the training program starting in June 2022, and the control arm will receive the training program after the final research data collection, with training from June 2023. If the trial is not able to recruit 63 women within the recruitment period, then the research team will consider an unequal allocation ratio with a smaller number of participants in the control group or extending data gathering time. The impact of this is discussed in the section on sample size.

## Methods

### Aim, design, and outcomes

This practical randomized trial aims to evaluate the impact of an educational program based on the ISD model on improving preventive behaviors of STI Iranian women. An exploratory sequential mixed-methods design will be used in the study.

### Outcome measures

The initial research questions addressed in this study are “Does the educational intervention based on Instructional System Design (ISD) model affect STI preventive behaviors among Iranian women?”. To answer this main question following primary outcomes are expected:

1. Determining and comparing the effects of an educational intervention designed based on the ISD model in STI knowledge among women in the target and control groups before, immediately one month, and three months after the interventions.
2. Determining and comparing the effects of an educational intervention designed based on the ISD model in STI vulnerability among women in the target and control groups before, immediately, one month, and three months after the interventions.
3. Determining and comparing the effects of an educational intervention designed based on the ISD model in STI prevention self-efficacy among women in the target and control groups before, immediately, one month, and three months after the interventions.
4. Determining and comparing the effects of an educational intervention designed based on the ISD model in STI prevention intentions among the target and control groups before, immediately, 1 month, and 3 months after the interventions.

Consequently, we expect the effect of our educational intervention on preventive action regarding STIs among women. To assess the expected secondary outcomes a self-reported assessment addressing preventive actions (using a condom, doing pap-test and genital examination) will be conducted.

### Ethical approval

This protocol and the template informed consent forms will be reviewed and approved by the sponsor and Medical Ethics Research Center of Tarbiat Modares University (reference: IR.MODARES.REC.1399.039). with respect to scientific content and compliance with applicable research and human subjects regulations. The protocol, site-specific informed consent forms (Persian language), participant education and recruitment materials, and other requested documents—and any subsequent modifications—also will be reviewed and approved by the ethical review bodies. Subsequent to the initial review and approval, Ethical Committees (TMU) will review the protocol at least annually. The Investigator will make safety and progress reports to the TMU at least annually and within six months of study termination. These reports will include the total number of participants enrolled . . . and summaries of each stage of intervention [33].

### Consent or assent

A trained research health educator will introduce the trial to participants who will receive a pdf file regarding the main aspects of the trial. participants will also receive information sheets. Trained Research will discuss the trial with participants in light of the information provided in the pdf file and information sheets. participants will then be able to have an informed discussion with the participating consultant. Trained Research will obtain written consent from participants willing to participate in the trial. Information sheets and consent forms are provided for all involved in the trial however these have been amended accordingly in order to provide separate information sheets and consent form. All information sheets, consent forms, and the pdf file are in the Persian language There are also separate information sheets and consent forms for the control group.

### Participants

The research population will include women aged 18–48 years who are sexually active. To meet these inclusion criteria, recruitment will be from women who refer to Tehran Municipality Health House Participants will be recruited from Tehran Municipality Health House affiliated by the Women Empowerment Headquarters of Tehran Municipality. The list of introduced eligible participants will be initially contacted via phone and given an in-depth description of the study, those interested will be followed up with an inclusion listing. Once Tehran Municipality Health House has received a briefing about the project and understands the randomized controlled trial design and data collection elements of the study, it will be asked to sign the consent form and allocate one room for the research project. Invitations to participate in the study will be stretched to all eligible women until we reach a sample size of 150 women who provide informed consent to participate. Table 1 contains a complete list of the inclusion and exclusion criteria.

**Table 1:** Inclusion and exclusion criteria

Inclusion criteria	Exclusion criteria
• Women aged 18–48 years • Women without cervical cancer • Women without mental disorders, drug dependence, and addiction • Tend to participate with informed consent to share information, and participate	• Absence of more than two sessions in training sessions • The participant has a special illness that is not able to participate in training sessions

### Study design

**Fig. 1:**
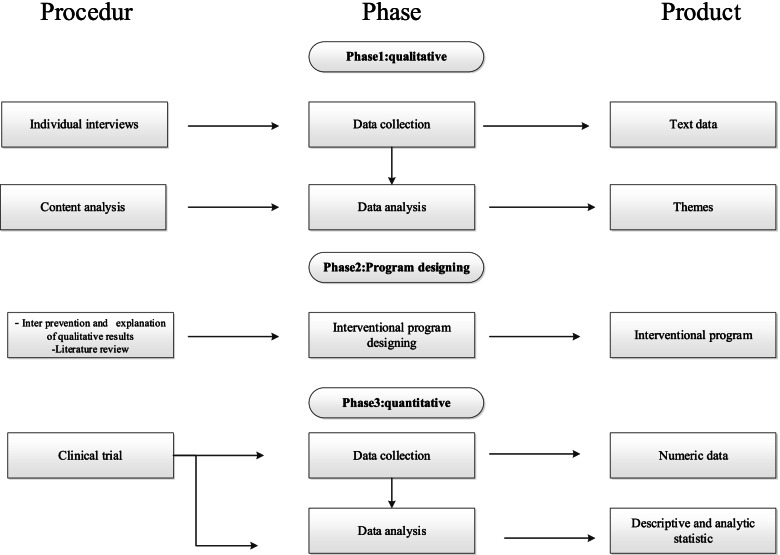
Study visual diagram

This exploratory sequential mixed-methods study will be divided into three phases, which are described below. Table 2 shows the enrollment, interview, intervention, and assessment schedule. This protocol was developed and reported in accordance with the Standard Protocol Items: Recommendations for Interventional Trials (SPIRIT), and the clinical trial will be carried out and reported in accordance with the Consolidated Standards of Reporting Trials (CONSORT). A visual diagram of the study process is shown in Fig. 1.

**Table 2:**
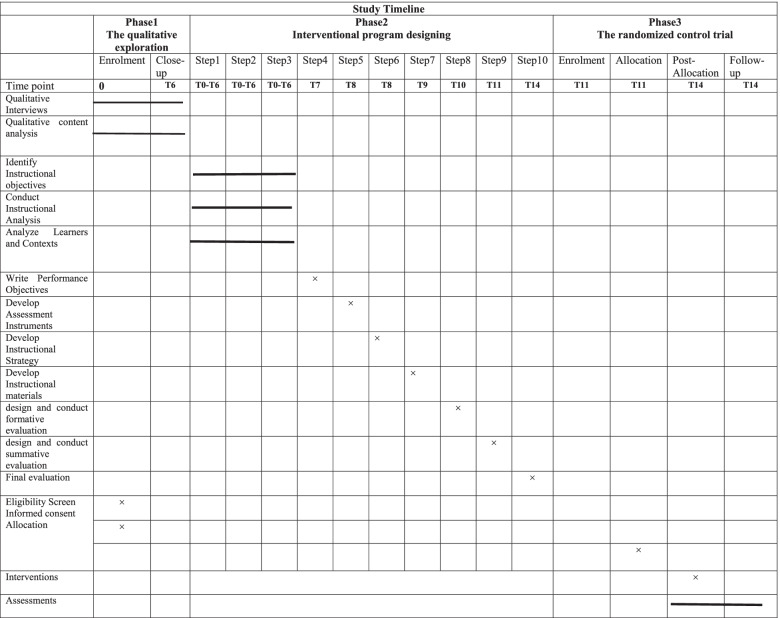
Schedule of enrolment, interviews, intervention, and assessment of the Educational Intervention trial, following the Standard Protocol Items Recommended for Clinical Trials (SPIRIT) guidelines

### Phase 1: the qualitative exploration

The qualitative study will take 5 months to complete. To explore the educational and learning needs of at-risk women, semi-structured interviews with open-ended questions and conventional qualitative content analysis methods will be used. The interviews will take place face-to-face in a mutually convenient quiet environment. In accordance with the study’s goals and objectives, an interview guide has been created. The first question is a wide, open-ended question about the participant’s feelings about STIs, to which they will be asked to respond in detail. Then, based on this response, further probing questions are asked. The goal is to gain a comprehensive understanding of women’s attitudes toward STIs. Each interview will be transcribed word by word immediately after each interview. The transcript will then be sent to each woman, along with a summary of key points extracted from each interview, to ensure that the interviewer has correctly interpreted their declarations (member checking), and any vague discrepancies will be resolved. In addition, we will use the literature review to identify the educational and learning needs of women about STIs in order to gain an understanding of their needs. Finally, an educational intervention that best fits the qualitative study results will be developed using the two approaches mentioned above (interview and literature review) and the ISD model.

### Phase 2: designing the intervention program

The second phase of the study begins after the required data has been collected through qualitative research. The purpose of this step is to develop an effective educational program based on the ISD model to promote preventive behavior from STIs among Iranian women.

### Conceptual framework of content development based on ISD model

Dick and Carey’s (2014) Instructional System Design (ISD) model provided the framework for this program. The ISD model depicts the processes and steps we take to effectively organize all components in order to achieve our objectives [34]. This model is a systematic and structured process that establishes a strong connection between stimulus (learning materials) and response (learning) [35]. The role of the environment in learning is highlighted in this systematic approach. According to this model, it is necessary to first identify the sub-skills that learners should master before selecting the stimulus and strategy that are appropriate for each sub-skill [36].

### Instructional System Design (ISD) model steps

#### The ISD includes 10 steps

1. Identify instructional goals
  The first stage determined what learners could accomplish after completing the educational process [37]. These objectives are the result of need assessments. Need assessments are analyses of the gap between one’s current status and one’s desired status.
2. Conduct instructional analysis
  When learners have achieved their instructional goals, what they should do is determined by stages in a hierarchy.
3. Analyze learners and contexts
  In this stage, we will analyze learners’ STI learning experience, preferences, traits, and learning situations.
4. Write performance objectives
  When students complete the education program, they develop a detailed action plan based on the knowledge they have gained, which is aligned with performance objectives.
5. Develop assessment instruments
  Assessment tools determine whether or not students met their objectives.
6. Develop instructional strategy
  The researcher determines the educational method that learners will use to achieve their ultimate learning goals during the educational strategy phase.
7. Develop instructional materials
  The researcher will choose educational materials at this stage based on the instructional strategy.
8. Design and conduct formative evaluation
  At this stage, the educational content will be made available to at least 5 to 10 women outside of the research team, and the content’s validity will be evaluated in terms of applicability, comprehensibility, simplicity, and attractiveness.
9. Design and conduct a summative evaluation
  Following the evaluators’ comments in Step 8, any possible and necessary corrections to the educational content will be made.
10. Final evaluation
  The effect of the intervention will be evaluated at this stage in three time periods: immediately, 1 month, and 3 months after the end of the educational intervention.

### Phase 3: the randomized controlled trial

**Fig. 2:**
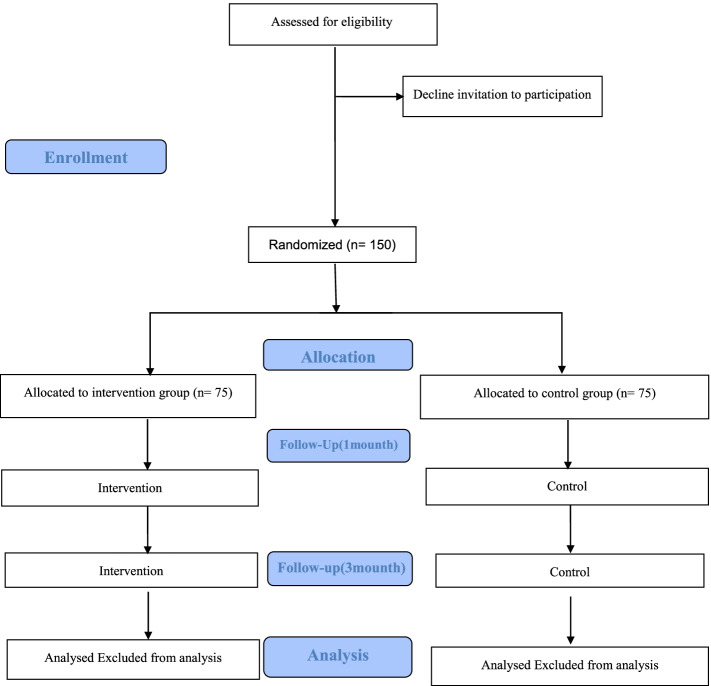
The flow chart of the randomized controlled protocol

Randomized controlled trials (RCTs) are the most effective way to assess public health interventions. RCT reduces the impact of confounding bias because each study participant is assigned to an intervention or control group solely by chance [38]. Figure 2 depicts the flow chart of the randomized controlled protocol.

### The intervention programs

The intervention will be created using the qualitative study’s phase 1 findings. Women who meet the inclusion criteria will be recruited indefinitely until the required sample size is reached. At this point, all participants will be coded and blindly allocated into one of the intervention and control groups by the researchers using a permuted block randomization program: (1) intervention (2) control.

Based on the design of the randomized controlled trial intervention, the impact of the intervention program on promoting women’s STI prevention behaviors will be assessed. Measurements will be taken at baseline, after one month, and after three months of following their respective program, as shown in Fig. 1. In accordance with ethical principles, the participants in the control group will also receive the most effective intervention after the final evaluation and comparisons of the three intervention groups.

### Strategies to improve adherence to interventions

Women are required to be present at the study site in person to receive the intervention, and any missed sessions are recorded to check adherence. To improve adherence, training program times are flexible between 9am and 5pm each day (room availability permitting), are not required to be at the same time of day, and are not required on weekends. Participants will be arranged for a free visit with a trained midwife for genital examination and health counseling.

### Instruments

The instrument that will be used to collect the data is the STI Four-Scale of Preventive Behaviors in females [2]. This scale was finalized by applying 40 five-point Likert response items where the items ranged from completely agree to completely disagree; the higher the score the greater the preventive STI behaviors. The calculated Content Validity Rate (CVR) and Content Validity Index (CVI) of the four-scale items ranged between 0.56–1.00 and 0.83–1.00, respectively. The impact score of all items was above 1.5. Cronbach’s alpha for each scale was as follows: STI knowledge (0.81), STI vulnerability (0.66), STI prevention self-efficacy (0.83), and STI prevention intentions (0.85). Cronbach’s alpha and intra-class correlation coefficient were calculated for the reliability of the four-scale items and ranged between 0.66–0.85 and 0.846–0.977, respectively.

### Sample size and power calculations

The sample size we require in the quantitative phase to provide sufficient power was calculated as 63 persons for each group. This sample size was calculated to be adequate at an alpha of 0.05 and a power of .80, to test for a difference between the groups. we considered a potential dropout rate of 30%. According to the formula below, we should start with a recruitment target of 75 participants in each group:
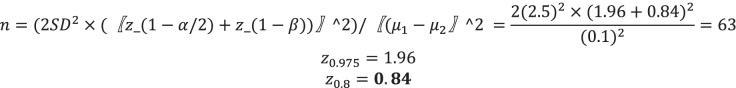


In accordance with this approach, a total of 150 women at risk, aged 18 to 48, from the Tehran Municipality Health House, will be recruited using the inclusion and exclusion criteria listed in Table 1.

### Randomization and blinding

After informed consent and baseline data collection, participants in the randomized controlled trial will be randomly assigned to the intervention and control arms in a restricted randomized block design. A research identification number will be given to each woman. Then, the identified individuals who volunteered to participate in the study were randomly assigned to the experimental and control groups using a blocking method. Following simple randomization procedures, participants will be assigned to one of two trial arms in a 1:1 ratio based on a computerized randomization program via https://www.sealedenvelope.com/simple-randomiser/v1. The letter A will be considered for the intervention group1 and the letter B for the control group. The process of random allocation will be continued continuously until the sample size will be reached. Masking of participants and study staff is not possible due to the nature of the intervention (educational) and allocation ratio. The first researcher (AKJ) will enroll participants in the study and allocate them a unique ID number. A statistic expert out of the research team (SHH) will generate the allocation sequence using the women’s numbers. The first researcher (AKJ) will then inform women which group they have been allocated to.

### Engagement and retention

As this is a group of participants that can be particularly difficult to participate and maintain in a study, given the sensitive nature of STIs, several steps will be taken to optimize retention. These include scheduling training at maximally convenient times for participants, hiring a trained midwifery that will be flexible and attentive to the individual’s needs, and frequent opportunities for questions and feedback. In addition, for a free visit, a trained midwifery will coordinate for a genital examination, and the names of the intervention group will be announced to the specialist.

### Data analysis

#### Phase 1 (qualitative analysis)

To carry out the qualitative content analysis process, each interview’s audio file will be listened to carefully several times on the same day and transcribed verbatim. In order to keep the data from the interviews private, each transcript will be assigned a code. To come up with the overall impression of the interviews and become fully immersed in the data, the audio files and transcripts will be reviewed several times, and any ambiguities and inconsistencies will be removed by comparing the audio files and the transcripts. The interviews will be audiotaped and a summary of the key points of each interview will be then sent to each participant to ensure that the interviewer will have interpreted that participant’s comments accurately [39]. The process of data analysis will be performed continuously and simultaneously with the data gathering process. All words, statements, and paragraphs that are relevant to the analysis process will be considered as a single semantic unit. After merging the semantic units, the codes will be extracted. The codes are combined to form subcategories, which are then combined to form the main categories. Finally, after the categories have been abstracted, the relevant themes will be identified [40]. The data will be managed using MAX.QDA-ver2020.

#### Phase 3 (quantitative analysis)

The collected data will be analyzed using descriptive statistics (such as frequency, frequency percentage, mean, and standard deviation) and inferential statistics in SPSS ver16. Generalized mixed models of analysis of variance for repeated measures will be used to compare the differences between the values obtained before the intervention and 1 and 3 months after the intervention in each group. We will also calculate the differences in means between the independent groups, as well as their respective 95% confidence intervals. All tests will be run with a 0.05 significance level (*p* 0.05). The Kolmogorov-Smirnov test will be used to determine the data’s normality.

#### Interim analysis

Intervention allocation will remain hidden for any interim analysis performed. Furturemore, As this is a minimal risk experiment, there will be no interim analysis or stopping instructions.

### Methods for additional analyses (non-adherence and missing data)

Missing data will be reported and links between results reviewed. depending on the extent of data missing an appropriate sensitivity analysis will be performed.

### Auditing

The auditors will follow a monitoring plan to verify that the clinical trial is conducted and that data are generated, documented, and reported in compliance with the protocol and the applicable regulatory requirements. Due to the nature of the educational intervention, there is no adverse event and no harm in this study. To our knowledge, the present study will not have any negative consequences. Every 6 months, we will send a report to the auditor. We will share the results of this study with keys Representative of the ethics committee and reviewers via presenting in related seminars.

### Protocol amendment

Any changes to the study protocol, including modifications in study objectives, study design, participants population, sample size, study procedure, or significant administrative aspects that may affect the conduct of the study, potential participants’ benefit, or participants’ safety will require protocol modification. Such amendments will be agreed upon by the Department of Health Education and Health Promotion, Faculty of Medical Sciences, Tarbiat Modares University, and will be approved by the Ethics Committee of Tarbiat Modares University prior to implementation. Administrative changes of the protocol are minor corrections and/or clarifications that have no effects on the way the study is to be conducted. These administrative changes will be agreed upon by Tarbiat Modares University. The Ethics Committee of the Faculty of Medical Sciences, Tarbiat Modares University may be notified of the administrative changes at the discretion of the Department of Health Education and Health Promotion, Faculty of Medical Sciences, Tarbiat Modares University.

## Discussion

This paper describes the clinical trial protocol which will examine intervention programs to promote preventive behaviors in at-risk women. This will be the first study to examine the impact of an educational program intervention based on ISD to promote preventive behaviors from STIs in at-risk Iranian women. Women in Iran account for a high percentage of the population. Despite the high prevalence of sexually transmitted infections affecting this community, low educational interventions have been made to enable them to preventive behaviors. Therefore, this study aims to identify the educational and learning needs of at-risk women and implement a purposeful intervention program as an effective step to promote preventive behaviors regarding STIs of Iranian women aged 18–48 years. This study has several robust design features detailed as follows:

1. Performing a randomized controlled trial based on the ISD model: This study is the first randomized controlled trial performed on women at risk of STIs to evaluate the effectiveness of the educational intervention.
2. Theory-based intervention: The gap in the majority of intervention studies is that they are designed based on educational needs. The present study explores the effectiveness of the training intervention over an educational framework called the Instructional system design (ISD) model.

We do recognize that this study will have some limitations in so far as we must limit the randomized controlled trial to women residing in one region of Iran. This, however, will enable us to maintain good control of the test procedure. There is a possibility of not having enough access to the questionnaire link and reducing the Response-Rate. To reduce this limitation, the link of the online questionnaire will be provided to the target group with maximum variance in different ways. Another limitation of this study is the possibility of samples falling in the final intervals of the study; It seems that considering the financial cost in exchange for completing the questionnaire in four stages (pre-test, one month later, and three months after the intervention) is a good solution. Last but not least, performing educational interventions in the field of sexual health among Iranian women always faces cultural problems. Accordingly, the issue of sexually transmitted diseases is no exception to this rule. Hence, it may be difficult to accept participants to enter the study at first.

## Conclusion

This study provides an educational program for educating, empowering, and promoting behaviors that prevent sexually transmitted infections. If the interventions designed in the present study are effective, It has a high practical potential for generalization for all women aged 18-48 at risk of STIs.

### Trial status

The study is ongoing. Recruitment opened in October 2020. The duration of the study period will be 2 years and will be finished in October 2022.

## Data Availability

Not applicable. The manuscript does not report data. The datasets subsequently generated and/or analyzed during the current study may be made publicly available following the conclusion of ongoing research. Requests for data may be made at any time to the corresponding author.

## Abbreviations

STIs: Sexually transmitted infections
ISD: Instructional System Design
RCT: Randomized controlled trial
CONSORT: Consolidated Standards of Reporting Trials
